# Comparative Patterns of Plant Invasions in the Mediterranean Biome

**DOI:** 10.1371/journal.pone.0079174

**Published:** 2013-11-14

**Authors:** Margarita Arianoutsou, Pinelopi Delipetrou, Montserrat Vilà, Panayiotis G. Dimitrakopoulos, Laura Celesti-Grapow, Grant Wardell-Johnson, Lesley Henderson, Nicol Fuentes, Eduardo Ugarte-Mendes, Philip W. Rundel

**Affiliations:** 1 Department of Ecology and Systematics, Faculty of Biology, National and Kapodistrian University of Athens, Athens, Greece; 2 Department of Botany, Faculty of Biology, National and Kapodistrian University of Athens, Athens, Greece; 3 Estación Biológica de Doñana, Consejo Superior de Investigaciones Cientificas, Sevilla, Spain; 4 Biodiversity Conservation Laboratory, Department of Environment, University of the Aegean, Mytilene, Greece; 5 Department of Environmental Biology, Sapienza University, Rome, Italy; 6 Curtin Institute for Biodiversity and Climate, School of Science, Curtin University, Bentley, Western Australia, Australia; 7 Agricultural Research Council–Plant Protection Research Institute, Pretoria, South Africa; 8 Facultad de Ciencias Forestales, Universidad de Concepción, Concepción, Chile and Institute of Ecology and Biodiversity, Santiago, Chile; 9 Departamento de Botánica, Universidad de Concepción, Concepción, Chile; 10 Department of Ecology and Evolutionary Biology, University of California, Los Angeles, California, United States of America; Lakehead University, Canada

## Abstract

The objective of this work was to compare and contrast the patterns of alien plant invasions in the world’s five mediterranean-climate regions (MCRs). We expected landscape age and disturbance history to have bearing on levels of invasion. We assembled a database on naturalized alien plant taxa occurring in natural and semi-natural terrestrial habitats of all five regions (specifically Spain, Italy, Greece and Cyprus from the Mediterranean Basin, California, central Chile, the Cape Region of South Africa and Southwestern - SW Australia). We used multivariate (hierarchical clustering and NMDS ordination) trait and habitat analysis to compare characteristics of regions, taxa and habitats across the mediterranean biome. Our database included 1627 naturalized species with an overall low taxonomic similarity among the five MCRs. Herbaceous perennials were the most frequent taxa, with SW Australia exhibiting both the highest numbers of naturalized species and the highest taxonomic similarity (homogenization) among habitats, and the Mediterranean Basin the lowest. Low stress and highly disturbed habitats had the highest frequency of invasion and homogenization in all regions, and high natural stress habitats the lowest, while taxonomic similarity was higher among different habitats in each region than among regions. Our analysis is the first to describe patterns of species characteristics and habitat vulnerability for a single biome. We have shown that a broad niche (i.e. more than one habitat) is typical of naturalized plant species, regardless of their geographical area of origin, leading to potential for high homogenization within each region. Habitats of the Mediterranean Basin are apparently the most resistant to plant invasion, possibly because their landscapes are generally of relatively recent origin, but with a more gradual exposure to human intervention over a longer period.

## Introduction

Biological invasions are impacting all components of ecological systems [1] and are contributing to biotic homogenization [2], [3]. Many alien species are now shared among floras and faunas that had evolved in isolation [3]. The increasing pace of species introductions [4] suggests an urgency to identify traits common to successful invaders, and habitat types more prone to be invaded. Species traits and habitat vulnerability to invasion are two pillars for effective risk assessment of biological invasions [5].

Certain habitats are highly vulnerable to plant invasions. Anthropogenic habitats exhibit higher levels of plant invasion as a result of higher propagule pressure and higher disturbance levels than natural or semi-natural habitats [6]. However, many alien plants also thrive in natural habitats [7]. Alien plants often invade fertile habitats with high water availability [7], especially after changes in disturbance regime [8]. To identify and investigate trends in habitat level of invasion, comparisons between similar climatic regions are informative as they reduce large-scale environmental variation [9], [10]. Climatically analogous regions are often influenced by analogous abiotic stressors and share homologous habitats. This allows analysis to focus on the effects of regional differences in the micro- and biotic environment [11], and on regional historical factors influencing the habitat-level of invasion. Nonetheless, a global synthesis of alien plant species characteristics and habitat vulnerability to invasions is missing for any single biome.

Mediterranean-climate regions (MCRs), namely the Mediterranean Basin, California, Central Chile, Cape Region of South Africa and Southwestern (SW) Australia, are commonly cited examples of convergent evolution in vegetation structure and function [12]. MCRs cover slightly more than 2% of the Earth’s land surface [13]; yet are home to about 20% of the world’s vascular plants, including many endemic species [13], and are all classified as global biodiversity hotspots [14]. Unfortunately, they are also expected to be among the world biomes likely to suffer greatest proportional changes in biodiversity as a result of the interactive effects of major drivers of global environmental change (i.e. climate and land use changes, [15]).

Preliminary insights have highlighted the importance of biological invasions in MCRs e.g. [16] and that diversity and ecosystem functions are increasingly threatened by introduced species in those regions [10], [17], [18]. Here, we explore the naturalized alien flora of an entire biome in a manner not previously attempted for any other biome (see [16]). We investigate similarities in the taxonomy, life-history traits, origin, and invaded habitats of naturalized neophytes in the five MCRs to answer the following questions: How similar is the taxonomic composition of the naturalized flora across regions? How do the naturalized taxa differ in their origin? What are the most common life history traits of the naturalized species? Which natural habitats display the highest and lowest numbers of alien plant species? And finally how similar is the taxonomic composition across habitats both within a region and across regions?

## Materials and Methods

### Areas of Study

**Figure 1:**
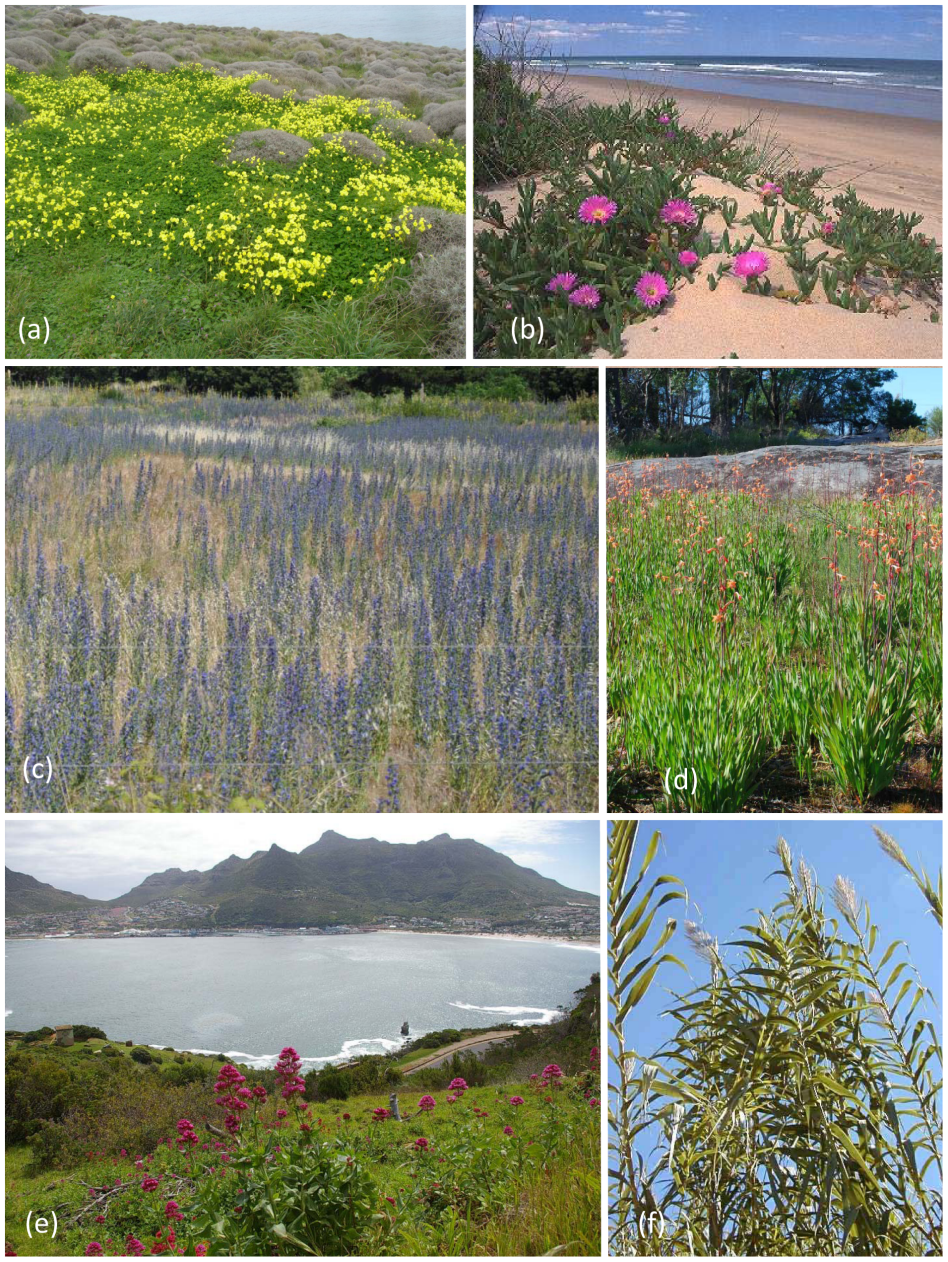
Examples of naturalized alien herbs and geophytes in the five mediterranean-climate regions. **1a**) The South African geophytic species *Oxalis pes-caprae* in Lesbos island, Greece, Mediterranean basin; **1b**) The South African succulent species *Carpobrotus edulis* in California, USA; 1c) The Eurasian biennial herbs *Echium plantagineum* and *E. vulgare* in Región del Maule, Central Chile; **1d**) The South African geophyte, *Watsonia meriana* var. *bulbillifera* on a granite outcrop in south-western Australia; **1e**) The Mediterranean basin region herb, *Centranthus ruber* in the Cape Region, South Africa; and **1f**) The tall Eurasian grass, *Arundo donax* in a Californian riparian system. Each of these species have become naturalised in multiple Mediterranean-climate regions. Photo credits Panayiotis Dimitrakopoulos **1a**; Philip Rundel **1b**, **1f**; Nicol Fuentes **1c**; Grant Wardell-Johnson **1d**; Lesley Henderson **1e**.

**Figure 2:**
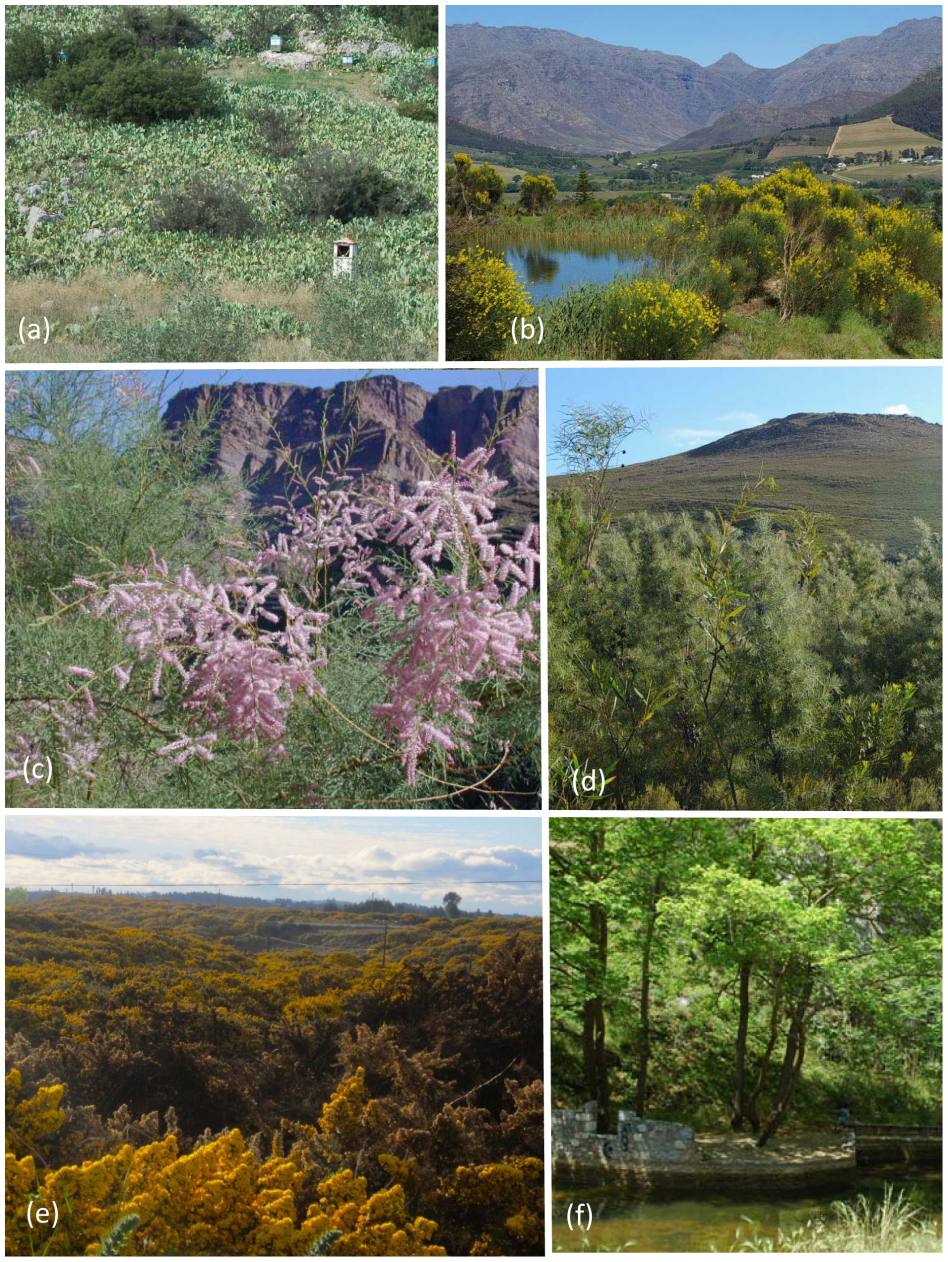
Examples of naturalised alien shrub and tree species in the five mediterranean-climate regions. **2a**) The southern USA succulent shrub *Opuntia ficus-barbarica* in abandoned grazed lands, Stylida, Greece, Mediterranean Basin; **2b**) The Mediterranean basin region shrub, *Spartium junceum* in the Cape Region, South Africa; **2c**) The central Eurasian tree *Tamarix ramossissima*, in coastal California; **2d**) Two Australian shrub species, *Hakea gibbosa* and *Acacia longifolia* in the Cape Region, South Africa; **2e**) The European spiny fabaceous shrub, *Ulex europaeus* in Chillán, Región del BioBio; and **2f**) The east Asian tree *Ailanthus altissima* in central Greece, Mediterranean Basin. Each of these species have become naturalised in multiple Mediterranean-climate regions. Photo credits Margarita Arianoutsou **2a**, **2f**; Lesley Henderson **2b**, **2d**; Philip Rundel **2c;** Jonathan Urrutia **2e**.

The areas of study are the world’s five MCRs, the Mediterranean Basin (a west-east gradient based on data from Spain, Italy, Greece and Cyprus), California, Central Chile, the Cape Region of South Africa and Southwestern (SW) Australia as described in Appendix S1. Figures 1 and 2 illustrate examples of invasive naturalized plant species within the five MCRs.

### Data Sources

Assembling a database on alien plant taxa from different sources included the challenge of establishing consistent criteria for compiling the appropriate taxa and habitat databases.The datasets used for analysis include only naturalized neophytes, i.e. alien plants introduced after 1500 AD which have established in the wild and do not need human assistance for population persistence [19]. These plants are hereafter referred as naturalized species. We have avoided the use of the term "invasive species" since it would apply to only part of the dataset. However, throughout the text the term level of invasion is used to indicate the number or density of alien species in a region or habitat. Selection of taxa to be included in analysis was based on the criterion that they should appear in natural or semi-natural habitats. The term “semi-natural habitats” recognises that disturbance associated with human activities has been a feature of MCRs, sometimes over considerable periods. While we do not consider highly modified human-made habitats such as dumping or waste places, street pavements and parks, we incorporate habitats with a history of disturbance, including SW Australian grasslands, which are self-perpetuating novel ecosystems resulting from the transformation of woodland to grassland. Some regional differences in data sources can be expected in this database, which includes species and life habit traits for an entire biome.

The Angiosperm Phylogeny Group [20], [21] system was adopted for classifying species into families. The species datasets were scrutinized for taxonomical discrepancies and complemented with data concerning growth form (grass, herb, subshrub/shrub, and tree), life form and life cycle (annual, biennial or perennial). Life forms were identified according to Raunkiaer’s system [22], which classifies vascular plants according to the place of the plant's growth-point (bud) during seasons with adverse conditions (cold or dry seasons).

Species were assigned to the habitat of occurrence in the introduced country, according to a classification system of the main vegetation types occurring across the five MCRs. The vegetation types were coded according to the EUNIS classification system level 1 (B: coastal habitats; C: inland wetlands; E: grasslands; F: shrublands; G: forests; H: rocks, screes, sparsely vegetated habitats) and second level subcategories. A full description of each vegetation type (hereafter habitat type) is provided in Appendix S2.

Native ranges were mainly assigned based on chorological categories (the geographical distributions of plants), as defined by Pignatti [23]. Species of hybrid origin include those which have arisen spontaneously through hybridization from at least one non-native parent species.

#### Mediterranean basin

The datasets used for the alien plants of the Mediterranean countries were those compiled for the DAISIE database (http://www.europe-aliens.org/) and updated with more recent data [24], [25], [26].

In this study, only taxa alien in all four countries representing the Mediterranean Basin were included for analysis. Those that are native to one country but alien in another were regarded as native and thus excluded from the dataset.

#### California

Data on naturalized species is derived from Baldwin [27], with naturalized plants defined as aliens growing in the wild or approximately wild conditions and reproducing either sexually or asexually. Alien taxa occurring outside cultivation but only in highly modified environments such as urban, suburban, or agricultural lands were removed from consideration.

#### Chile

Data for Chile were retrieved from a database which includes naturalized species (*sensu* Richardson et al. [19]) occurring in the Mediterranean-climate zone of the country (30°–38° southern latitude). We included specimens in CONC and SGO herbaria (details and list in [28]) and the more recent compilation of naturalized species [29].

#### Cape region of south africa

The sources of data were the Southern African Plant Invaders Atlas (SAPIA) database [30], as well as [31] and [32]. The SAPIA database is a computerized catalogue of some 70,000 locality records of more than 600 naturalized plant species in Southern Africa. The database incorporates records gathered by about 560 participants in the SAPIA mapping project since 1994 and from roadside surveys conducted by Henderson since 1979. Herbaceous species are under-represented in this database largely as a consequence of biased recording of the larger, more conspicuous species during roadside surveys. Data source [31] lists the most important environmental weeds in the South African study area (the fynbos), while [32] kept the alien species in their conspectus of the Cape Flora to a minimum and included only those which have become naturalized and might be mistaken for part of the native flora.

#### Southwestern australia

For southwestern Australia, all naturalized species were selected that were recorded in, but not restricted to the nine Mediterranean-climate bioregions [33], based on multiple occurrence of records in the WA herbarium (http://florabase.calm.wa.gov.au/weeds/).

### Data Analysis

Matrices of species presence/absence in each region and in each habitat-per region were generated. Hierarchical clustering and ordination (non-metric multi-dimensional scaling NMDS) were performed on the ranked Bray-Curtis’s similarity matrix calculated on the species presence – absence data of the taxa [34]. This approach allowed non parametric comparisons between datasets to show trends, and groupings of similar assemblages, along with the variables describing the associated environment (e.g. [35]). The South African habitat types of ‘coastal rocks’ (Br), ‘grasslands’ (E) and ‘sparsely vegetated harsh habitats’ (H), hosting only 1, 2 and 3 species respectively were omitted from the data pool. All analyses were performed with the PRIMER ® 6.1.4 software [36].

## Results

### Taxonomic Similarity across Regions

A total of 1627 naturalized species were recorded in the natural and semi-natural habitats of the five MCRs (Table 1). The species’ database includes 38 taxa at subspecies level, 26 taxa at variety level and 39 hybrids. In total, 18 species are represented by more than one infraspecific taxon. However, for simplicity we refer to all taxa with the term “species”. These species belong to 705 genera and 147 families.

**Table 1:** Number of naturalized species for each of the five mediterranean-climate regions (a), number of species occurring in more than one of the five regions with indication of their percentage in parenthesis (b), and pooled number of species (c).

	(a) Total per region						(b) Shared							(c) Total
Taxa	California	Chile	Med. Basin	South Africa	SW Australia		California		Chile	Med. Basin	South Africa	SW Australia		all regions
Families	107	45	85	59		109	96 (90)	44 (98)		74 (87)	57 (97)		95 (87)	14
Genera	368	203	231	134		424	262 (71)	181 (89)		159 (69)	117 (87)		292 (69)	705
Species & infraspecific taxa	617	349	414	174		791	301 (49)	248 (71)		178 (43)	114 (66)		384 (49)	1627
Species per area (10^3^ km^2^ )	1.9	2.3	0.4	1.9		2.6								

Areas in 10^6^ km^2^ (Cowling et al. 1996): California = 0.32; Chile = 0.14; Mediterranean Basin = 0.93 (for the 4 countries studied); South Africa = 0.09 SW Australia = 0.31.

The geographical distribution of the five MCRs included in this study is depicted in citation [13].

**Figure 3:**
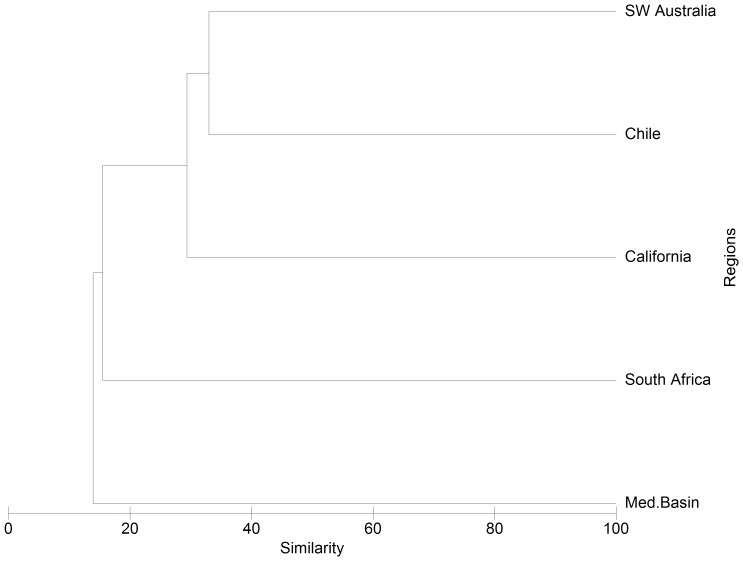
Naturalized alien species similarity across the five regions of the mediterraean biome.

Richness of naturalized species and genera was highest in SW Australia, and lowest in South Africa. SW Australia had the highest number of naturalized species present per unit area followed by Chile, South Africa and California, and the Mediterranean Basin had the lowest. At the species level, percentages of shared taxa were markedly higher in Chile and South Africa than in the other three regions (Table 1). A similar but less distinct trend was noted in the numbers of shared genera and families. All regions shared a higher percentage of species with SW Australia than with any other region (Table 2). Chile shared, along with the Mediterranean Basin, comparatively less species with the other regions (only c. 8–24%). The only species common to all five regions were the South American *Nicotiana glauca* (Solanaceae), the grass *Paspalum vaginatum* and three woody legumes - the Northern American *Robinia pseudoacacia* and the Eastern Australian *Acacia dealbata* and *A. melanoxylon*. Hierarchical clustering confirmed the low similarity in species composition of the naturalized floras across the five regions (Fig. 3). The highest similarity was found between Chile and SW Australia (∼ 33%) and between this group and California (∼29%). The Poaceae was the most frequently represented family with a large number of naturalized species in all regions, ranging from 10% (Mediterranean Basin) to 20% (Chile) of the total species (Appendix S3). The next most frequent families were the Asteraceae, in all regions except in South Africa, and the Fabaceae. Otherwise, comparatively high frequencies were also noted for certain families in a subset of regions, notably Amaranthaceae and Onagraceae in the Mediterranean Basin, Myrtaceae in South Africa, and Iridaceae in SW Australia.

**Table 2:** Pair-wise numbers of shared naturalized species in the five mediterranean-climate regions of the world.

	California	Chile	Med. Basin	South Africa	SW Australia
**California**	**316** (51)	146 (24)	71 (12)	60 (10)	201 (33)
**Chile**	146 (42)	**101** (29)	35 (10)	41 (12)	188 (54)
**Med. Basin**	71 (17)	35 (8)	**236** (57)	41 (10)	113 (27)
**South Africa**	60 (34)	41 (24)	41 (24)	**60** (34)	76 (44)
**SW Australia**	201 (25)	188 (24)	113 (14)	76 (10)	**407** (51)

Numbers in bold correspond to the number of species existing only in the assigned region. Numbers appearing in parentheses are percentages of the total number of aliens of the country in the same row.

### Native Origins

Naturalized species occurring in the Mediterranean Basin originate mostly from North and/or South America, although species of Asian and African origin are also prominent (Table 3). The naturalized species in South Africa originated primarily from America and Australasia and secondarily from Eurasia and Africa. On the other hand, the naturalized flora of SW Australia, Chile, and California includes higher numbers of African and Eurasian than American and Australasian aliens. The African (mainly South African) component is prominent in SW Australia and the Eurasian in Chile and California.

**Table 3:** Number of naturalized plant species in the five mediterranean-climate regions according to their native origin.

Region	California	Chile	Med. Basin	South Africa	SW Australia	All Regions
Africa	50 (8)	11 (3)	44 (11)	5 (3)	177 (22)	226 (14)
Asia	51 (8)	6 (2)	63 (15)	13 (7)	26 (3)	121 (7)
Asia, Africa	3 (0)	1 (0)	2 (0)	1 (1)	9 (1)	12 (1)
Eurasia	79 (13)	67 (19)	15 (4)	12 (7)	84 (11)	165 (10)
Eurasia, Africa	116 (19)	76 (22)	(0)	25 (14)	95 (12)	181 (11)
Europe	33 (5)	74 (21)	1 (0)	5 (3)	41 (5)	111 (7)
Europe, Africa N	11 (2)	6 (2)	(0)	2 (1)	14 (2)	21 (1)
Mediterranean	31 (5)	17 (5)	2 (0)	3 (2)	70 (9)	90 (6)
Mediterranean & regions*	44 (7)	25 (7)	(0)	6 (3)	44 (6)	67 (4)
North and/or South America	125 (20)	44 (13)	226 (55)	41 (24)	136 (17)	414 (25)
Australasia	38 (6)	6 (2)	22 (5)	41 (24)	37 (5)	95 (6)
Africa/Europe/Asia**, America	8 (1)	3 (1)	2 (0)	4 (2)	13 (2)	20 (1)
Africa/Europe/Asia**, Australasia	9 (1)	2 (1)	2 (0)	2 (1)	(0)	13 (1)
Tropics/Subtropics	8 (1)	5 (1)	14 (3)	2 (1)	13 (2)	28 (2)
Cosmopolitan/Sub-Cosmopolitan	5 (1)	2 (1)	2 (0)	1 (1)	6 (1)	13 (1)
Other	3 (0)	(0)	(0)	(0)	3 (0)	5 (0)
Cultivated/Hybrid	1 (0)	(0)	10 (2)	1 (1)	12 (2)	22 (1)
Uncertain/Unknown	2 (0)	4 (1)	9 (2)	10 (6)	9 (1)	21 (1)

Numbers in parenthesis are percentages within a region.

* *Mediterranean Basin and one region in Europe or Asia or N. Africa.*

** *Regions in Africa and*/or *Europe and/or Asia.*

### Life History Traits

The majority of the alien species recorded exhibited an herbaceous growth form (Table 4). Trees were the least frequent growth form, except in South Africa where they represented 39% of its naturalized flora. The Mediterranean Basin had a comparatively high percentage of shrubs (26%). Regarding life span, the perennial cycle was the most prominent in three of the regions; however annuals in SW Australia and Chile comprised 45 and 54% of the flora, respectively. Phanerophytes and therophytes had equally high frequencies in the Mediterranean Basin, while phanerophytes were by far the most frequent life form in South Africa (53%). Hemicryptophytes represented the highest frequency in California (38%), followed by therophytes (31%).

**Table 4:** Life history traits of naturalized plant species for each of the five mediterranean climate regions.

Life history trait		California	Chile	Med. Basin	South Africa	SW Australia
**Growth Form**	Herb	504 (82)	324 (93)	266 (64)	77 (44)	652 (82)
	Shrub	69 (11)	19 (5)	106 (26)	30 (17)	103 (13)
	Tree	44 (7)	6 (2)	42 (10)	67 (39)	36 (5)
**Life cycle**	Annual	192 (31)	176 (50)	120 (29)	20 (11)	280 (35)
	Annual/Biennial	12 (2)	16 (5)	1 (0)	(0)	41 (5)
	Annual/Perennial	26 (4)	8 (2)	3 (1)	2 (1)	68 (9)
	Biennial	16 (3)	13 (4)	7 (2)	1 (1)	7 (1)
	Perennial	371 (60)	136 (39)	279 (68)	151 (87)	395 (50)
**Life form**	Therophyte	192 (31)	187 (54)	118 (29)	21 (12)	357 (45)
	Therophyte/other	33 (5)	23 (7)	4 (1)	1 (1)	25 (3)
	Hemicriptophyte	232 (38)	89 (26)	78 (19)	32 (18)	24 (3)
	Hemicryptophyte/other	0 (0)	6 (2)	2 (0)	1 (1)	4 (1)
	Geophytes	13 (2)	9 (3)	37 (9)	9 (5)	160 (20)
	Hydrophyte	34 (6)	(0)	24 (6)	9 (5)	14 (2)
	Chamaephyte	22 (4)	12 (3)	28 (7)	9 (5)	121 (15)
	Phanerophyte	91 (15)	23 (7)	119 (29)	92 (53)	86 (11)

Numbers in parenthesis are percentage values within a region.

### Habitat Level of Invasion

With the exception of SW Australia, naturalized species established mainly in relatively few habitat types (Table 5). Across all five regions, the highest numbers of species were recorded in inland wetlands, riparian woodlands (and shrublands) and grasslands. The least invaded habitats were coniferous forests, coastal rocks, coastal wetlands and short, open shrublands. Riparian woodlands hosted the majority of the alien plants of South Africa and SW Australia, and a comparatively high percentage in the Mediterranean Basin and California. The percentage of aliens in coastal sand and shingle was highest in Chile. Tall, dense shrublands hosted a comparatively high number of aliens in SW Australia and South Africa, and less so in Chile. Deciduous and broadleaved forests and sparsely vegetated habitats hosted a high number of aliens only in SW Australia.

**Table 5:** Number of naturalized species invading each habitat category identified in the five mediterranean-climate regions.

Habitat type	California	Chile	Med. Basin	South Africa	SW Australia	TOTAL
Coastal rocks (Br)	no data	25 (7)	20 (5)	1 (1)	101 (13)	**173**
Coastal sand and shingles (Bs)	49 (8)	142 (41)	98 (24)	21 (12)	262 (33)	**440**
Coastal wetlands (Bw)	28 (5)	no data	43 (10)	16 (9)	106 (13)	**176**
Inland wetlands (C)	186 (30)	212 (61)	210 (51)	66 (38)	512 (65)	**832**
Grasslands (E)	190 (31)	201 (58)	176 (43)	2 (1)	462 (58)	**721**
Short open shrublands (F)	100 (16)	no data	21 (5)	41 (24)	-	**153**
Tall thick shrublands (Fm)	no data	75 (21)	21 (5)	45 (26)	236 (30)	**334**
Deciduous and broadleaved forests (G)	81 (13)	6 (2)	33 (8)	10 (6)	469 (59)	**542**
Coniferous forests (Gc)	53 (9)	3 (1)	24 (6)	–	–	**79**
Riparian woodlands and shrublands (Gw)	140 (23)	43 (12)	183 (44)	112 (64)	569 (72)	**728**
Sparsely vegetated harsh habitats (H)	31 (5)	32 (9)	37 (9)	3 (2)	179 (23)	**260**
**TOTAL**	**617**	**349**	**414**	**174**	**791**	

Totals represent the total number of species in each country and in each habitat. Percentages (in brackets) are calculated on the basis of the total number of plants in the last row. Empty cells denote that there are no alien species records but habitat category occurs in the region; dash (–) denotes that the habitat category is absent from the region.

For Australia details are given in Appendix S1 with habitat categories.

Many alien species occur in multiple habitats. Thus, 60% of species occurred in more than one habitat type and 28% in 4–11 habitat types. The occurrence in multiple habitats is less frequent in the Mediterranean Basin, South Africa and California (56%, 63% and 69% of the aliens in only one habitat type, respectively), and highly frequent in SW Australia (25% of aliens in only one habitat type). Moreover, in SW Australia 39% of the aliens occur in 4 or more habitat types in contrast to 3–16% in all other regions.

The growth form spectra of alien species differed among habitats across regions (Table 6), with the herbaceous growth form dominant in all habitat types except short shrublands and conifer forests. Here shrubs and trees were more prominent. Although all growth forms occurred in all habitats, the majority of herbaceous species occurred in inland wetland habitats (57%), while riparian woodlands hosted the majority of shrubs (46%) and a notable 74% of trees.

**Table 6:** Number of naturalized species occurring in each habitat category identified in the five mediterranean-climate regions, regardless of the region.

Habitat type	Herb	Shrub	Tree	Total plants/habitat
Coastal rocks	134 (11)	40 (15)	13 (10)	**187**
Coastal sand and shingle	362 (29)	74 (28)	25 (18)	**461**
Coastal wetlands	152 (12)	23 (9)	11 (8)	**186**
Inland wetlands	718 (57)	89 (34)	45 (33)	**852**
Grasslands	619 (49)	107 (41)	20 (15)	**746**
Short open shrublands	77 (6)	55 (21)	37 (27)	**169**
Tall thick shrublands	253 (20)	56 (21)	42 (31)	**351**
Deciduous and broadleaved forests	444 (35)	80 (30)	44 (32)	**568**
Coniferous forests	47 (4)	18 (7)	17 (13)	**82**
Riparian woodlands	536 (42)	121 (46)	101 (74)	**758**
Sparsely vegetated harsh habitats	202 (16)	56 (21)	13 (10)	**271**
**Total plants/growth form**	**1269**	**263**	**136**	

Percentages (in brackets) are calculated on the basis of the total number of plants in the last row. Note that the same taxon might appear as different growth form in each region and this explains the difference in total plants per habitat compared to Table 5.

### Species Similarity across Habitats

**Figure 4:**
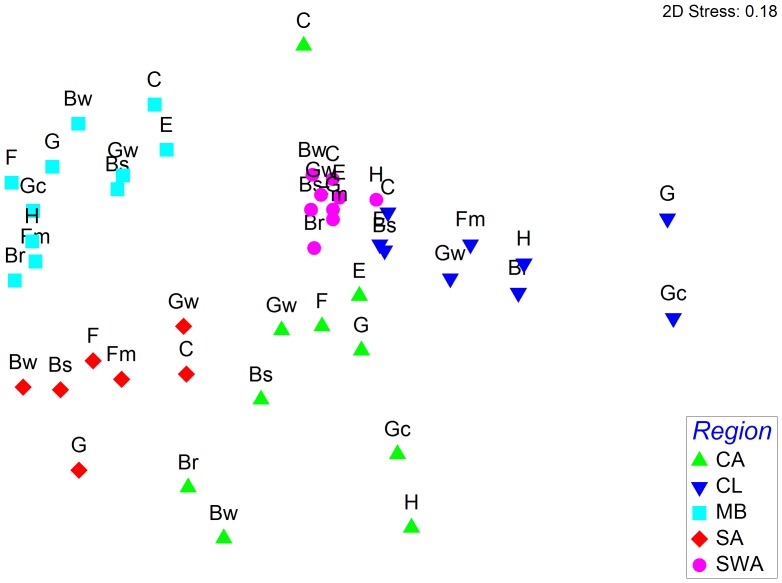
Non-metric multidimensional scaling ordination (NMDS), (stress = 0.18) showing taxonomic differences in naturalized species among habitats in each of the five mediterranean-climate regions (MCRs). CA: California, CL: Chile, MB: Mediterranean Basin; SA: South Africa; SWA: SW Australia. See Table 5 for explanation of habitat acronyms.

Habitats were more similar by region than by type (Fig. 4). That is, naturalized species assemblages are more homogeneous among different habitats within a region than among the same habitat type in different regions. There are two major regional groups (similarity <5%), whose members are loosely connected: the habitats of SW Australia and Chile (similarity ∼10%) and the habitats of the Mediterranean Basin and South Africa (similarity <10%), with the Californian habitats placed between them.

The highest homogeneity among species assemblages among habitats within a region was observed in SW Australia, where habitat types had a similarity level higher than 35%. The habitats of Chile also present high homogeneity in species assemblages (similarities 25–58%), with the exception of forests (G, Gc) which are separated due to the few species hosted. Habitat types of California, by contrast, host the least homogeneous species assemblages (10–20% similarity).

**Figure 5:**
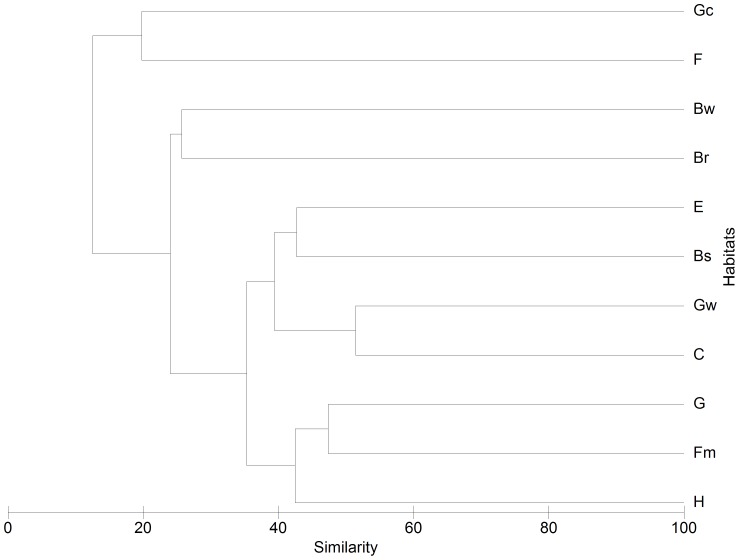
Dendrogram (Bray Curtis metric, UPGMA) showing similarity of mediterranean-climate regions habitats based on the basis of naturalized species between habitat types regardless of region. See Table 5 for explanation of habitat acronyms.

The analysis of similarity of species assemblages among habitats separated two groups with high (∼40%) similarity levels (Fig. 5). The first group includes some of the most invaded habitats in all regions (i.e. coastal sands, inland wetlands, riparian woodlands and grasslands). The second group includes habitats with fewer species (i.e. deciduous forests, tall shrub and sparsely vegetated habitats).

## Discussion

This study has for the first time, compared the identity and extent of invasion of natural and semi-natural habitats by naturalized plant species for an entire biome, providing new insights about patterns of global biotic homogenization [37].

Herbaceous perennials were the most frequent taxa, with SW Australia exhibiting both the highest numbers of naturalized species and the highest taxonomic similarity among habitats, and the Mediterranean Basin the lowest. Low stress and highly disturbed habitats had the highest frequency of invasion and homogenization in all regions, and high natural stress habitats the lowest, while taxonomic similarity was higher among different habitats in each region than among regions. Habitats of the Mediterranean Basin were apparently the most resistant to plant invasion, possibly because their landscapes are not in general ancient [38], [39], and because they have had a more gradual exposure to human intervention over a longer period [40], [41].

### Taxonomic Similarity across Regions

The Poaceae, Asteraceae (except South Africa), and Fabaceae were the most species-rich families in all regions. A similar result was found in a world-wide analysis [42]. These are among the largest families of vascular plants, are cosmopolitan in distribution, and include many taxa of importance to agriculture, horticulture and the world economy. Moreover, they possess species with traits permitting them a high level of habitat penetration. These traits include high reproductive rate, high grazing resistance (Asteraceae) or resilience (Poaceae) as well as increased dispersal efficiency (Poaceae and Fabaceae) and nitrogen fixation ability (Fabaceae) [43], [44].

SW Australia had the highest number of naturalized species (level of invasion) at all taxonomic levels and also the highest naturalized species density. Conversely, the Mediterranean Basin had the lowest density of naturalized species. Several hypotheses may be advanced to explain these regional differences which cannot be solely attributed to variation in area [45]. Geographic and landscape heterogeneity may increase both native and non-native species richness, and may have a stronger positive effect on the latter [46]. However, SW Australia lacks the topographic diversity of the other regions.

Fire enhances invasion in plant communities in many areas of the world [47]. In MCRs fire has shaped patterns of plant diversity [38], [48], except in Chile [49]. Deviations from the natural fire regime may be a factor explaining a high density of aliens in Chile where pre-human fire frequency was very low [49]. In Chile native herbaceous species do not recruit following fire as in other Mediterranean regions [50]. Regarding the Mediterranean Basin, there is little evidence of fire being the trigger for plant invasions [51].

Levels of invasion are usually positively related to human population density, urbanization and agricultural land use as surrogates of habitat disturbance and propagule pressure [6], [52]. These factors alone only partly explain our results. SW Australia has the lowest population density and urban area, while the Mediterranean Basin and California have the highest [53]. On the other hand, SW Australia has the highest percentage of total agricultural land, but California the lowest [53].

Differences in agricultural land use in relation to landscape age and biogeography [38], [39] may hold the key. Mediterranean regions differ in their landscape age, climatic and disturbance history. Much of the landscape in SW Australia and South Africa has been long without disturbances that remove all biotic material from an area (i.e.*Tabula rasa* disturbances – after [54]). Prolonged leaching and erosion of ancient soils result in low levels of major soil nutrients, with a high proportion of species being maladapted in high nutrient conditions [38], [39]. A large proportion of the ancient landscape of SW Australia has recently been converted to agriculture [39], requiring continuing fertilizer input to remain productive. These agricultural landscapes, are then self-perpetuating, with little return of native taxa, even after decades of fallow [55], contrasting with relatively young landscapes [38], [39] with moderate fertility soils [49], [56] such as Chile, California and many areas of the Mediterranean basin.

Because naturalized species are most prevalent in fertile soils [6], nutrient enrichment makes low fertility soils vulnerable to invasions [57]. Thus, in the presence of new species-pools – both from nutrient-rich (e.g. Mediterranean Basin) and nutrient-poor areas (South Africa), the environments of SW Australia are particularly vulnerable to naturalized plant establishment in the presence of disturbance and changed land use.

The Mediterranean Basin has been exposed to continuous tectonic activity and climatic vicissitudes, especially since the last glaciations, and has also been affected by high-density human activities since pre-historic times [56]. It has been suggested that plant species in the Mediterranean Basin have a pre-adaptation to human disturbance that both helps native communities withstand invasions, and have the potential to become particularly invasive in other regions [56]. Moreover, the persistence of traditional forms of agriculture such as terraced fields facilitates the recovery of the native vegetation and prevents the establishment and spread of invasive species [40]. These factors may also explain the markedly low level of invasion observed in Mediterranean Basin shrublands. The situation is different in the other MCRs. Chile and California have both been affected by tectonic events and glaciation, but to a lesser extent than the Mediterranean Basin, and have been colonized relatively recently by Euroamericans. In South Africa, despite the long presence of humans, major landscape modification had been limited until the 20th century. In Australia by contrast, the arrival of indigenous people at least 40,000 ago was far-reaching in its impact on wildlife and on landscape processes, particularly in relation to fire. However, few introduced species accompanied this transformation. European colonization in both South Africa and Australia was responsible for recent landscape transformation through deforestation for agriculture, livestock introduction, and urbanization [56]. European colonization was also accompanied by the introduction of vast numbers of plant species (and animals) for forestry, agriculture, and horticulture.

### Native Origins

Species similarity patterns are apparently related to differences in the origin of the naturalized species, since American and Australasian taxa were prominent in Africa and Eurasia and vice-versa (Table 4). The origin is, in turn, apparently related to the history of introduction. In the Mediterranean Basin introductions from the neighboring European areas, northern Africa and western Asia are generally classified as archaeophytes, while neophytes are mostly American, Asian and African species. Several historical episodes could be important in explaining these biogeographical patterns (see [24] and references therein). The relatively similar patterns of species origin in California and Chile are related to the Spanish colonization, first in Chile (mid-1500s) and later in California (late 1700s), which led to landscape alterations and alien species introductions [58]. In addition, immigration (from Asia and Europe) and international trade was intensified in California relative to Chile since the 1850’s, enhancing biotic exchange [9].

In South Africa, the naturalized species originated primarily from America, Australasia and Africa, while those in Australia have originated primarily from Africa, America and Eurasia (Table 3). Colonization of SW Australia by northern Europeans since 1826, and the stopover of ships in South African ports on route to Australia prior to the opening of the Suez Canal may have led to many early introductions [59] in both South Africa [41] and SW Australia. However, by far the majority of introduced taxa to SW Australia were the result of agricultural development during the 20^th^ century and of horticultural introductions beginning in the 19^th^ Century. A few important aliens in both South Africa and Australia were the result of forestry development [60].

### Plant Life History Differences among Regions

The single general pattern that has emerged for all regions was the high frequency of the herbaceous growth form and, except in Chile, the perennial life cycle (Table 5). However, life form patterns were different among regions. The perennial life cycle provides the potential for clonal growth, a trait correlated with alien abundance (e.g., [61]). On the other hand, the increased frequency of woody phanerophytes in the Mediterranean Basin and South Africa is probably due to agroforestry and horticultural introductions [24], [62]. In South Africa, in particular, tree invasions mainly involve Australian *Acacia* and *Hakea* species, and northern hemisphere *Pinus* species [62] extensively planted for dune stabilization and for timber [63].

Annuals represented a significant percentage in all regions except South Africa, and were dominant in Chile. This may be related to their ability to avoid the summer drought stress, typical in MCRs, which is also a reason for their abundance in the native floras [64]. However, annuals are abundant in the alien floras of other climate regimes as well [65], [66]. Hence their large numbers may also be related to other factors such as their frequent occurrence as ruderals [67] and success in disturbed habitats, or their frequent inadvertent introduction through contaminated stock, seed and ballast. European non-native annual grassland species in California, Chile and SW Australia arrived mainly in the period of European settlement [9], [56] and in Californian grasslands they have replaced both native perennials [68] and annuals, possibly as a response to grazing [69]. In Chile, the high percentage of annuals may be a further result of anthropogenic fires which have increased the presence of alien annuals in natural shrublands. This increase can be attributed to both the large seedbanks of alien annuals, and their increased propagule pressure relative to native taxa [70].

There were more differences than similarities in the life history trait spectra across the five regions. Possible explanations for these patterns might be context dependent, partly related to regional introduction histories. Studies in Mediterranean areas have found no significant association of longevity, growth form or life form to invasion success [71]. The general failure to identify global features of successful invaders is in line with suggestions that the outcome of invasions is highly idiosyncratic [72]. Moreover, there are indications that the ecological attributes of successful invaders are habitat-dependent [72]. It has been suggested that alien invasion is facilitated by the absence of native species in the same functional group [73], [74], but this has not been confirmed in the Mediterranean islands [75].

### Patterns of Habitat Invasion

Overall, a common pattern in all regions is that inland wetlands and riparian woodlands are amongst the habitats with the highest numbers of naturalized species, while conifer forests, coastal wetlands, coastal and inland rocks and deserts are the habitats with the lowest levels of invasion. The high level of invasion in riparian and wetland habitats is also a common finding in other biomes (e.g. [7]), and has been attributed to greater anthropogenic and recurrent natural disturbance which result in fluctuating nutrient availability, and high propagule pressure [76]. Moreover, the reduced drought stress in these habitats is believed to increase the vulnerability to invasions [77]. Fluctuating resources may also account for increased numbers of aliens in dry grasslands and coastal sands in most regions. These are the habitats subject to highest anthropogenic disturbance, causing vegetation openings and elevated nutrient levels. Conversely, non-native annual grasses dominate most Californian mediterranean grasslands except those on serpentine habitats with low productivity, which act as refugia for native species [78]. This is considered a result of their abiotic resistance to invasion [79]. In a similar mode, the low invasion level of conifer forests and of rock and desert habitats may be related to low levels of resource availability and anthropogenic disturbance in combination with high drought and temperature stress. Low invasion levels in conifer forests have also been observed in other regions (e.g. [80]). The high level of invasion in cliff habitats found in other studies [80], [81] may be due to their pooling with city walls and quarries. Notably, comparatively higher number of species in rocky habitats of SW Australia may be associated with these sites having a high annual native species pool in a low nutrient environment. Circumstances of nutrient enrichment and seasonally bare habitats combined with new species pools render these sites vulnerable to naturalization. By comparison, coastal wetlands include low numbers of naturalized species, and are characterized by naturally high productivity and resource fluctuations.

### Taxonomic Similarity across Habitats and within Regions

Alien species assemblages had higher taxonomic similarity between different habitats within a region than within the same habitat in different regions. This has also been observed among European regions with different climates [80], and among large Mediterranean Basin islands [82]. The highest alien taxonomic similarity between regions occurred in SW Australia where the same cohort of alien species is able to establish in different habitats [83]. The highest number of alien species occurring in multiple habitats was observed in SW Australia, and the lowest in California. In California the alien flora of pairs of counties had a differentiating effect in half of the comparisons, and taxonomic similarity increased with increasing distance between counties [84]. However, because taxonomic similarity is scale-dependent (e.g. [85]), a decrease in the spatial scale of sampling area (e.g. at the community level; [86]) could produce important differences among the same habitat type across regions.

## Conclusions

The plant species that have successfully naturalized in the five mediterranean – climate regions very often occur in multiple habitats, leading to potentially high homogenization of the floras within each region. By their very nature these taxa often have traits of great dispersal ability, rapid growth, and capacity to colonize following disturbance. It remains to be explored whether differences in the level of invasion among habitats are due to differences in propagule pressure or to differences in invasibility. Further exploration of whether differences in the susceptibility to invasion are due to biotic or abiotic constraints to alien species establishment and survival is therefore required.

Our results suggest that the terrestrial habitats of the Mediterranean Basin are the most resistant to the occurrence of naturalized plant species among the five MCRs. Although this statement has been put forward before [16], it is the first time that it has been based on a quantitative analysis which encompasses data from all five MCRs. Our analysis of the large databases across the regions supports two hypotheses. The first is associated with the geological and climatic history of the regions [38], [39], and the second with human interactions with these very different geological and climatic histories [40], [58]. The ancient landscapes (predominately) in the South African and SW Australian MCRs have rendered them particularly susceptible to the addition of nutrients and new species pools. At the same time, the incorporation of agricultural and other land management practices (including new patterns of disturbance) from the very different landscapes of Europe has facilitated the widespread naturalization of an alien flora. Fortunately, significant areas remain unaltered by nutrient additions. Management attention should be directed towards ensuring that these areas remain safe havens for the extraordinary biota of these regions. As anthropogenic environmental change provides increasing conservation dilemmas, attention to naturalized species, disturbance and refugia [87] will become of increased significance in the conservation of the floras of these regions.

## Supporting Information

Appendix S1 **{Description of study areas}.**
(DOC)

Appendix S2 **{Definition of habitat types}.**
(DOC)

Appendix S3 **{Number of taxa per family in the five mediterranean-climate regions}.**
(DOC)
