# Supplementary material for: Comparative Patterns of Plant Invasions in the Mediterranean Biome
Source: PLoS One. 2013 Nov 14;8(11):e79174. doi: 10.1371/journal.pone.0079174 (PMC3828305; doi:10.1371/journal.pone.0079174)
Supplement: Appendix S1 — {Description of study areas}. (DOC) [file pone.0079174.s001.doc]

# **Appendix S1.** Description of study areas.

# **Mediterranean Basin**

Four countries represent the Mediterranean Basin in this work, Spain, Italy, Greece, and Cyprus that are representative of a well-defined east-west gradient. A substantial proportion of their territory is covered by mountains and hills, while their insular character is important especially for Greece and Italy. Biogeographical factors, geological history, landscape heterogeneity, along with the history of human presence and impact mostly contributed to the richness of their present-day biodiversity. These countries contain all the main habitats found in the Mediterranean Basin. Increased human population concentrations mostly in coastal areas, land-use changes, pollution and the introduction of alien species are of the major drivers of changes in their biodiversity. A more detailed description of the specific study areas and analysis of their alien flora can be found in Arianoutsou *et al.,* (2010).

# **California**

The California Floristic Province (CFP) as generally defined approximates the political boundaries of the state, extending into southern Oregon and northwestern Baja California, but excluding the desert areas. The region is topographically and climatically diverse. The dynamic geological history of uplift, faulting, and tectonics has produced complex mosaics of soil structure and parent material, and produced sharp climate shifts over the Quaternary. Typical mediterranean-type vegetation is a mix of evergreen shrubland (chaparral), drought-deciduous shrublands, evergreen and deciduous oak woodlands, and grasslands. Also present are mesic coastal forests along the northwest coast and extensive high elevation mountains with montane and subalpine coniferous forests. California as a political unit contains about 4900 native vascular plants species, with about 4000 of these present within the state’s area of the CFP. Invasive species within the areas of the CFP in Oregon and Baja California are not included here but these add relatively few taxa.

**Chile**

The Mediterranean region in Chile occupies a narrow band from 30 to 38°S covering around 145,000 km^2^. This ecosystem represents the transition between the driest deserts in the world (Atacama Desert north of 28°S), and the mixed deciduous-evergreen forests (south of 36°S) (Luebert & Pliscoff, 2006). The area is characterized by a longitudinal depression trenched between two mountain ranges: the Coastal Cordillera and the Andes. These ranges are separated by a narrow tectonic basin, the 80-100 km wide Central Depression. The climatic regime of the area differs in some important aspects from other Mediterranean ecosystems of the world— the absence of summer rainfall and associated thunderstorm activity (Armesto *et al.,* 2007).

The Mediterranean landscape and natural vegetation (*matorral*) in Chile has been strongly transformed by human activities (logging, burning, grazing, etc.); over 11 million inhabit this area (c. 80% of total in Chile), (Arroyo *et al*., 2000). Since the Spanish colonization 500 years ago, many plants and animals were progressively introduced (Arroyo *et al*., 2000). These changes in landscape coincided with the widespread removal of natural vegetation cover for agriculture and forestry plantations (i.e., *Pinus* and *Eucalyptus* spp.) (Arroyo *et al*., 2000). Natural vegetation currently consists of clumps of sclerophyllous shrubs and trees, surrounded by a seasonal herbaceous matrix dominated by alien plants, most of which originated from the Mediterranean Basin (Arroyo *et al*. 2000; Montenegro *et al*. 1991). An area of 104,000 km^2^ in the Mediterranean zone has 2395 native species (2332 angiosperms, 6 gymnosperms, and 57 pteridophytes) in 591 genera (Arroyo *et al*. 1995).

**South Africa**

Mediterranean climate vegetation prevails over most of the Cape Floristic Region, situated at the south-western tip of Africa, and covers an area of approximately 90,000 km^2^. The climate is strictly Mediterranean in the west, but receives substantial summer precipitation in the eastern half. The region includes an estimated 9,000 native species of vascular plants, 69% endemic. The largest families are the Asteraceae and Fabaceae, together comprising some 20% of the total species. The flora is dominated by evergreen, fine-leaved sclerophyllous shrubs, very few trees and a remarkably large number of geophytes (herbaceous perennials with bulbs, corms or rhizomes). The main vegetation types are heathland and shrubland, locally called fynbos and renosterveld (Goldblatt & Manning, 2000). Fynbos typically occurs on nutrient-poor sandstone soils and renosterveld on richer, fine-grained soils. Fynbos is most similar to kwongan in Australia both of which are distinguished from shrublands in other Mediterranean climate regions by their relatively open shrub cover, high shrub species diversity, and the highest incidence in the world of obligate post-fire re-seeders, canopy seed storage (serotiny), and ant seed dispersal (myrmecochory) (Cowling *et al.*, 1996).

**South-western Australia**

The Mediterranean-climate region of south-western Australia (SW Australia) occupies about 304,700 km^2^, and is isolated from the rest of the Australian MCR by ocean and arid lands. This region includes over 7,300 native vascular plant taxa (36% of Australia’s vascular plant taxa) with high levels of endemism (49% for vascular plants) and over 2,500 plants of conservation concern (Hopper & Gioia, 2004). The region experiences dry, warm summers and cool, wet winter gradients of increasing temperature and decreasing rainfall occurring from both South to North, and west to east. Virtually the entire vegetation of the region is naturally dominated by woody plants. However, large areas of woodlands and lesser areas of forest have been deforested for agriculture and are now managed principally for cropping and livestock production. Areas on ancient landscapes of the region, once cleared for cropping and grazing have become self-perpetuating grasslands, even in the absence of agriculture. The south-west high rainfall zone includes tall open-forests (to 80 m high), open-forest, woodlands and heaths. *Eucalypt* woodlands and *Acacia* shrublands occur in the east and north of the region. Mallee (a shrub growth form exhibited by many eucalypt species) and proteaceous shrub-heaths (kwongan) occur on residual lateritic uplands and derived sandplains.

**REFERENCES**

Arianoutsou, M., Delipetrou, P., Celesti-Grapow, L., Basnou, C., Bazos, I., Kokkoris, Y., & Vilà, M. (2010) Comparing naturalized alien plants and recipient habitats across an east-west gradient in the Mediterranean Basin. *Journal of* *Biogeography*, **37**, 1811–1823.

Armesto, J.J., Arroyo, M.T.K., & Hinojosa, L.F. (2007) The mediterranean environment of Central Chile. *The Physical Geography of South America* (ed. by T.T. Veblen, K.R. Young and A.R. Orne), pp. 184-199. Oxford University Press, New York.

Arroyo, M.T.K., Cavieres, L., Marticorena, C. & Muñoz-Schick, M. (1995) Convergence in the Mediterranean floras in central Chile and California: Insights from comparative biogeography. *Ecology and Biogeography of Mediterranean Ecosystems in Chile, California, and Australia* (ed. by M.T.K. Arroyo, P.H. Zedler and M.D. Fox), pp. 43-88. Springer-Verlag, New York.

Arroyo, M.T.K., Marticorena, C., Matthei, O. & Cavieres, L. (2000) Plant invasions in Chile: present patterns and future predictions. *Invasive species in a changing world* (ed. by H.A. Mooney and R.J. Hobbs), pp. 43-88. Springer-Verlag, New York.

Cowling, R.M., Rundel, P.W., Lamont, [B.B](http://www.sciencedirect.com/science/article/pii/0169534796100446#AFF3%23AFF3)., Arroyo, M.K. & Arianoutsou, M. (1996) Plant diversity in Mediterranean-climate regions. *Trends in Ecology and Evolution*, **11**, 362-366.

Goldblatt, P. & Manning, J. (2000) Cape Plants. A Conspectus of the Cape Flora of South Africa. *Strelitzia* 9, National Botanical Institute of South Africa, Pretoria & Missouri Botanical Garden, St Louis.

Hopper, S.D. & Gioia, P. (2004) The south-west Australian Floristic Region: evolution and conservation of a global hot spot of biodiversity. *Annual Review of Ecology,* *Evolution and Systematics*, **35**, 623-650.

Luebert, F. & Pliscoff, P. (2006) *Sinopsis bioclimática y vegetacional de Chile*. Editorial Universitaria. Santiago de Chile.

Montenegro, G., Teillier, S., Arce, P. & Poblete, V. (1991) Introduction of plants into the mediterranean-type climate of central Chile. *Biogeography of Mediterranean invasions* (ed. by R.H. Groves and F. di Castri), pp. 103-114. Cambridge University Press, Cambridge.
