# Supplementary material for: Comparative Patterns of Plant Invasions in the Mediterranean Biome
Source: PLoS One. 2013 Nov 14;8(11):e79174. doi: 10.1371/journal.pone.0079174 (PMC3828305; doi:10.1371/journal.pone.0079174)
Supplement: Appendix S2 — {Definition of habitat types}. (DOC) [file pone.0079174.s002.doc]

**Appendix S2.** Definition of habitat types and symbol used for their coding. The EUNIS classification Level 1 was used for coding the broad categories B, C, E, F, G, and H.

| **Habitat type** | **Habitat Definition** | **Code** |
| --- | --- | --- |
| Coastal rocks | All types of coastal rocks and rocky habitats | Br |
| Coastal sand and shingle | All types of sand dune habitats, including dune wetlands and also shingle beaches | Bs |
| Coastal wetlands | Mainly halophytic coastal wetlands (e.g. salt marshes) and also fresh-water coastal wetlands (California) | Bw |
| Inland wetlands | Mainly fresh-water inland wetlands, including wet grasslands and also inland salt lakes (SW Australia) | C |
| Grasslands | Dry grasslands, including synanthropic grasslands. SW Australian grasslands are the result of deforestation and are not included. | E |
| Short open shrublands | Low, cushion-shaped scrub or low shrubland with few tall shrubs, includes chaparral and sage scrub (California), Renosterveld (S Africa), phrygana (Mediterranean Basin) | F |
| Tall thick shrublands | Mediterranean tall and thick shrub (maquis), fynbos (S Africa) and all heath/shrubland of SW Australia | Fm |
| Deciduous and broadleaved forests | All types of deciduous and broadleaved evergreen forests, including California oak woodlands and the eucalypt tall forests to open woodlands of SW Australia. | G |
| Coniferous forests | All types of conifer forests. Conifer forests in SW Australia are plantations and not included. | Gc |
| Riparian woodlands | Riparian forests and shrublands | Gw |
| Sparsely vegetated harsh habitats | Inland rocks and screes and inland desert (only California) | H |
