# Supplementary material for: Comparative Patterns of Plant Invasions in the Mediterranean Biome
Source: PLoS One. 2013 Nov 14;8(11):e79174. doi: 10.1371/journal.pone.0079174 (PMC3828305; doi:10.1371/journal.pone.0079174)
Supplement: Appendix S3 — {Number of taxa per family in the five mediterranean-climate regions}. (DOC) [file pone.0079174.s003.doc]

**Appendix S3.** Number of taxa per family in the five mediterranean-climate regions of the world: CA = California, CL = Chile, MB = Mediterranean Basin, SWA = Southwestern Australia.

| **Family (APG classification)** | **CA** | **CL** | **MB** | **SA** | **SWA** | **Total** |
| --- | --- | --- | --- | --- | --- | --- |
| Poaceae | 108 | 71 | 43 | 26 | 135 | 236 |
| Asteraceae | 78 | 55 | 53 | 5 | 73 | 177 |
| Fabaceae | 33 | 40 | 20 | 23 | 78 | 122 |
| Brassicaceae | 30 | 13 | 8 | 2 | 35 | 62 |
| Amaranthaceae | 15 | 10 | 28 | 5 | 19 | 54 |
| Iridaceae | 6 |  | 6 |  | 46 | 54 |
| Rosaceae | 20 | 6 | 16 | 7 | 16 | 50 |
| Cyperaceae | 21 | 5 | 12 | 2 | 15 | 44 |
| Caryophyllaceae | 19 | 18 |  | 1 | 24 | 42 |
| Polygonaceae | 21 | 11 | 14 | 1 | 15 | 41 |
| Solanaceae | 3 | 8 | 17 | 5 | 19 | 36 |
| Onagraceae | 5 | 1 | 23 | 2 | 13 | 33 |
| Lamiaceae | 20 | 7 |  | 1 | 15 | 29 |
| Plantaginaceae | 17 | 12 | 3 | 2 | 12 | 29 |
| Apiaceae | 5 | 11 | 8 | 2 | 11 | 26 |
| Myrtaceae | 10 |  | 2 | 15 | 7 | 26 |
| Euphorbiaceae | 5 | 6 | 9 | 2 | 10 | 24 |
| Aizoaceae | 10 | 1 | 9 |  | 8 | 21 |
| Boraginaceae | 8 | 9 | 3 |  | 5 | 21 |
| Cactaceae | 3 |  | 17 | 3 | 1 | 20 |
| Geraniaceae | 8 | 8 | 1 |  | 10 | 18 |
| Juncaceae | 10 |  | 1 |  | 9 | 16 |
| Malvaceae | 5 | 5 | 4 | 1 | 9 | 16 |
| Scrophulariaceae | 6 | 2 | 3 | 1 | 9 | 16 |
| Convolvulaceae | 4 | 1 | 6 | 4 | 8 | 15 |
| Crassulaceae | 5 |  | 2 |  | 8 | 13 |
| Ranunculaceae | 7 | 5 |  | 1 | 2 | 11 |
| Salicaceae |  | 2 | 4 | 6 | 2 | 11 |
| Agavaceae | 1 |  | 5 | 3 | 4 | 10 |
| Hydrocharitaceae | 6 | 1 | 6 | 1 | 2 | 10 |
| Oxalidaceae | 3 | 1 | 3 |  | 8 | 10 |
| Araceae | 5 |  | 2 | 2 | 3 | 9 |
| Plumbaginaceae | 6 |  |  | 1 | 3 | 9 |
| Rubiaceae | 5 | 3 |  |  | 5 | 9 |
| Apocynaceae | 4 | 1 | 4 | 3 | 7 | 8 |
| Hyacinthaceae |  |  |  |  | 8 | 8 |
| Pinaceae |  |  | 3 | 5 | 4 | 8 |
| Verbenaceae |  | 3 | 5 | 1 | 5 | 8 |
| Alliaceae | 1 |  | 2 |  | 6 | 7 |
| Amaryllidaceae | 1 |  |  |  | 6 | 7 |
| Hypericaceae | 6 | 3 | 2 |  | 2 | 7 |
| Asparagaceae | 1 |  | 1 |  | 5 | 6 |
| Asphodelaceae | 4 |  | 1 |  | 2 | 6 |
| Fumariaceae | 1 | 3 |  |  | 3 | 6 |
| Oleaceae | 3 |  | 1 | 2 | 2 | 6 |
| Papaveraceae |  | 3 |  |  | 6 | 6 |
| Sapindaceae | 2 |  | 3 | 1 | 2 | 6 |
| Alismataceae | 2 | 2 | 2 |  | 2 | 5 |
| Anacardiaceae | 3 |  | 2 | 3 | 2 | 5 |
| Arecaceae | 3 |  | 3 | 2 | 2 | 5 |
| Balsaminaceae | 3 |  | 3 |  | 1 | 5 |
| Cucurbitaceae |  |  | 1 |  | 4 | 5 |
| Gentianaceae | 1 |  |  |  | 5 | 5 |
| Linaceae | 2 | 2 |  |  | 2 | 5 |
| Lythraceae | 5 | 1 |  |  | 1 | 5 |
| Orobanchaceae | 2 | 5 |  | 1 | 4 | 5 |
| Vitaceae | 1 |  | 3 |  | 2 | 5 |
| Bignoniaceae | 1 |  | 2 |  | 1 | 4 |
| Fagaceae | 1 |  | 2 | 1 |  | 4 |
| Molluginaceae | 4 |  |  |  |  | 4 |
| Moraceae | 3 |  | 1 | 1 | 2 | 4 |
| Passifloraceae | 2 |  |  | 1 | 2 | 4 |
| Pontederiaceae | 3 |  | 2 | 2 |  | 4 |
| Primulaceae | 2 | 2 |  | 2 | 3 | 4 |
| Salviniaceae | 3 |  | 2 | 1 | 1 | 4 |
| Adiantaceae |  | 1 |  | 2 |  | 3 |
| Araliaceae | 2 |  |  |  | 2 | 3 |
| Cannabaceae |  |  | 1 |  | 2 | 3 |
| Cannaceae |  |  | 1 | 1 | 2 | 3 |
| Caprifoliaceae |  |  | 3 |  | 1 | 3 |
| Dipsacaceae | 2 | 2 |  |  | 1 | 3 |
| Haloragaceae | 2 |  | 2 | 2 | 1 | 3 |
| Juglandaceae | 2 |  | 1 |  |  | 3 |
| Nyctaginaceae | 1 |  | 2 |  | 1 | 3 |
| Orchidaceae | 1 |  |  | 1 | 1 | 3 |
| Phytolaccaceae | 1 |  | 1 | 1 | 1 | 3 |
| Proteaceae |  |  |  | 3 |  | 3 |
| Resedaceae | 1 |  |  |  | 3 | 3 |
| Tamaricaceae | 3 |  |  | 1 | 1 | 3 |
| Ulmaceae | 2 |  | 1 |  |  | 3 |
| Urticaceae | 1 | 2 |  |  | 2 | 3 |
| Violaceae |  | 2 | 1 |  | 1 | 3 |
| Acanthaceae | 1 |  |  |  | 1 | 2 |
| Betulaceae |  | 1 | 1 | 1 |  | 2 |
| Campanulaceae |  |  |  |  | 2 | 2 |
| Casuarinaceae |  |  | 1 | 1 | 2 | 2 |
| Commelinaceae | 1 |  | 1 | 2 |  | 2 |
| Dennstaedtiaceae |  |  |  |  | 2 | 2 |
| Elaeagnaceae | 1 |  | 2 |  |  | 2 |
| Ericaceae | 1 |  |  |  | 1 | 2 |
| Hydrophyllaceae |  |  | 1 |  | 1 | 2 |
| Martyniaceae |  |  | 1 |  | 1 | 2 |
| Nymphaeaceae | 2 |  |  |  | 2 | 2 |
| Polygalaceae |  |  |  |  | 2 | 2 |
| Portulacaceae |  | 1 |  |  | 2 | 2 |
| Pteridaceae | 1 |  |  | 1 |  | 2 |
| Rhamnaceae |  |  | 1 |  | 1 | 2 |
| Rutaceae |  | 1 |  |  | 1 | 2 |
| Tetragoniaceae | 1 | 1 | 1 |  | 1 | 2 |
| Valerianaceae | 1 |  |  | 1 | 2 | 2 |
| Zingiberaceae | 1 |  |  | 1 |  | 2 |
| Zygophyllaceae |  |  | 1 |  | 1 | 2 |
| Actinidiaceae |  |  | 1 |  |  | 1 |
| Agapanthaceae |  |  |  |  | 1 | 1 |
| Alstroemeriaceae |  |  |  |  | 1 | 1 |
| Aponogetonaceae | 1 |  |  |  |  | 1 |
| Aquifoliaceae | 1 |  |  |  |  | 1 |
| Aristolochiaceae |  |  |  |  | 1 | 1 |
| Azollaceae |  |  | 1 | 1 |  | 1 |
| Basellaceae |  |  | 1 |  | 1 | 1 |
| Berberidaceae | 1 |  |  |  |  | 1 |
| Cabombaceae | 1 |  |  |  |  | 1 |
| Calceolariaceae |  | 1 |  |  |  | 1 |
| Celastraceae | 1 |  |  |  |  | 1 |
| Cleomaceae |  |  | 1 |  |  | 1 |
| Cupressaceae |  |  | 1 |  |  | 1 |
| Cytheaceae |  |  |  |  | 1 | 1 |
| Davalliaceae |  |  |  | 1 | 1 | 1 |
| Dryopteridaceae | 1 |  |  |  | 1 | 1 |
| Ebenaceae |  |  | 1 |  |  | 1 |
| Elatinaceae | 1 |  | 1 |  |  | 1 |
| Frankeniaceae | 1 |  |  |  | 1 | 1 |
| Hemerocallidaceae |  |  | 1 |  |  | 1 |
| Hydrangeaceae |  |  | 1 |  |  | 1 |
| Juncaginaceae |  |  |  |  | 1 | 1 |
| Lauraceae | 1 |  |  | 1 | 1 | 1 |
| Linderniaceae |  |  | 1 |  |  | 1 |
| Meliaceae | 1 |  | 1 | 1 |  | 1 |
| Melianthaceae |  |  |  |  | 1 | 1 |
| Menyanthaceae | 1 |  |  |  |  | 1 |
| Musaceae |  |  |  |  | 1 | 1 |
| Nelumbonaceae |  |  | 1 |  |  | 1 |
| Pittosporaceae |  |  |  | 1 | 1 | 1 |
| Platanaceae |  |  | 1 |  | 1 | 1 |
| Polemoniaceae | 1 |  |  |  |  | 1 |
| Potamogetonaceae | 1 |  |  |  |  | 1 |
| Punicaceae |  |  |  | 1 |  | 1 |
| Sapotaceae |  |  | 1 |  |  | 1 |
| Sarraceniaceae | 1 |  |  |  |  | 1 |
| Saururaceae |  |  | 1 |  |  | 1 |
| Simaroubaceae | 1 |  | 1 |  | 1 | 1 |
| Taxaceae |  |  |  |  | 1 | 1 |
| Taxodiaceae |  |  | 1 |  |  | 1 |
| Tecophilaeaceae |  |  |  |  | 1 | 1 |
| Tropaeolaceae | 1 |  | 1 |  | 1 | 1 |
| Typhaceae |  |  |  |  | 1 | 1 |
| Zosteraceae | 1 |  |  |  |  | 1 |
